# Classical and lectin complement pathways and markers of inflammation for investigation of susceptibility to infections among healthy older adults

**DOI:** 10.1186/s12979-020-00189-7

**Published:** 2020-06-10

**Authors:** David C. LaFon, Steffen Thiel, Young-il Kim, Mark T. Dransfield, Moon H. Nahm

**Affiliations:** 1 Division of Pulmonary, Allergy, and Critical Care, University of Alabama at Birmingham, Birmingham, AL USA; 2 UAB Lung Health Center, Birmingham, AL USA; 3 Department of Biomedicine, Aarhus University, Aarhus, Denmark; 4 Division of Preventive Medicine, University of Alabama at Birmingham, Birmingham, AL USA; 5 Birmingham VA Medical Center, Birmingham, AL USA; 6 Department of Microbiology, University of Alabama at Birmingham, Bevill Building Room 614 (BBRB 614), 845 19th Street South, Birmingham, AL 35294 USA

**Keywords:** Complement system, Lectin, Immune, Inflammation, Aging, Elderly

## Abstract

**Background:**

There is increasing recognition of the significance of chronic, low-level inflammation in older adults, or “inflammaging.” Innate immune responses and host-bacterial interactions are recognized as key factors in inflammaging. Inflammatory cytokine IL-6, and complement protein C1q have been identified as biomarkers for the development of frailty and aging-related diseases. Older adults are also susceptible to infections with serotypes of *Streptococcus pneumoniae* that bind ficolin-2, a component of the lectin complement pathway, and low ficolin-2 levels could possibly be involved in such susceptibility.

**Methods:**

The aim of our study was to evaluate complement pathway components and biomarkers for inflammaging among older adults in order to investigate potential innate immune mechanisms that may account for susceptibility to infections in this population. We compared inflammatory markers, as well as components/activity of the classical and lectin complement pathways between healthy older and younger adults. We hypothesized that older adults would have higher levels of inflammatory markers and C1q, and lower levels of lectin pathway components. Older (≥70 years old) and younger (19–54 years old) adults without significant smoking history or chronic medical conditions were eligible for participation. Inflammatory markers (IL-6, TNF-α, CRP), classical complement pathway activity (CH50) and protein levels (C1q, C3, C4), and lectin pathway (MBL levels/activity, CL-L1, MASP-1/2/3, MAp44, MAp19, and H/M/L-ficolin) were compared between groups.

**Results:**

Older adults had significantly higher mean levels of IL-6 and TNF-α. There were no significant differences in lectin pathway components between older and younger adults. Unexpectedly, mean C1q was significantly higher in the younger group in both unadjusted and adjusted analyses. There was also a significant association between race and C1q levels, but this association did not completely account for the observed differences between age groups.

**Conclusions:**

We did not observe deficiencies in lectin pathway components to account for increased susceptibility to ficolin-binding serotypes of *S. pneumoniae*. Elevated levels of inflammatory cytokines in older adults are suggestive of inflammaging. However, the observed age and race-associated changes in C1q have not been previously reported in the populations included in our study. These findings are relevant to the investigation of C1q in aging-related pathology, and for its proposed role as a biomarker for frailty and disease.

## Background

**Fig. 1:**
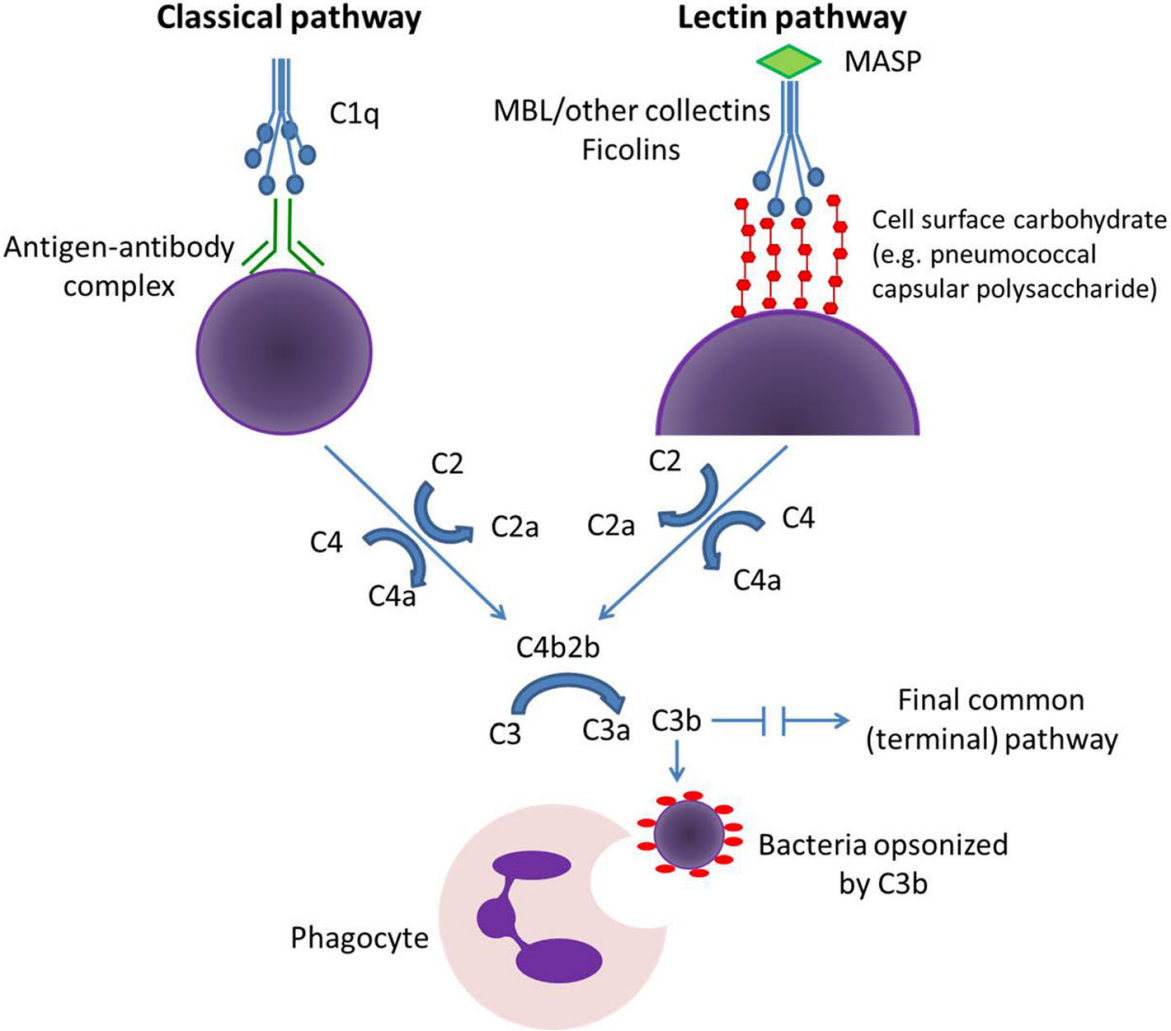
Classical and lectin pathways of complement activation [5]. The classical pathway of complement activation (top left) is initiated by the binding of C1q to antigen-antibody complexes. In contrast, the lectin pathway (top right) is initiated when collectins (including mannose-binding lectin, or MBL) or ficolins, in association with MBL-associated serine proteases (MASPs), bind to targets on cell surfaces. Both pathways, along with the alternative pathway (not shown), ultimately result in formation of C3b, which opsonizes pathogens, and anaphylatoxins C5a and C3a, and results in clearance via phagocytosis (bottom center) or through lysis of membranes via the terminal pathway

The world is currently experiencing a rapid and dramatic increase in the population of older adults [1]. An aging global population has spurred interest in the investigation of longitudinal physiologic and health-related changes in this group. Low-level, chronic inflammation with aging, or “inflammaging,” has been identified as an important factor in the development of frailty in older adults, and has been associated with the pathogenesis and progression of chronic disease [2–4]. Associations between frailty and disease and inflammation/immunity are of sufficient strength to allow for identification of pro-inflammatory cytokine interleukin-6 (IL-6) and complement proteins C3 and C1q (Fig. 1) as biomarkers for the development of frailty and poor prognosis [4, 6]. Host-bacterial interactions, including innate immune responses to commensal bacterial pathobionts, have been recognized as important contributors to chronic inflammation in older adults [2]. Therefore, measurement of elevated levels of inflammatory markers may reflect chronic immune responses to bacteria.

Innate immune responses may also contribute to increased risk of infections among older adults. An important consequence of aging-related immune dysfunction is increased susceptibility to acute bacterial infections, particularly infections caused by *Streptococcus pneumoniae*, or the pneumococcus. *S. pneumoniae* is a commensal pathobiont that inhabits the nasopharynx, and can cause severe infections in older adults. Pneumococcal infections are a major cause of morbidity and mortality among the elderly, and older adults have much higher rates of pneumococcal infections as compared with younger adults [7]. Risk for pneumococcal disease does not correspond with levels of pneumococcal IgG antibodies among older adults, as it does among children and younger adults [8, 9]. The specific mechanisms that account for these observations are incompletely understood, however they do suggest that innate immune responses may be an important component of susceptibility to pneumococcal infections among the elderly. The lectin pathway of the complement system can be particularly significant in immune responses to encapsulated bacteria such as pneumococcus, since it may be initiated by binding of the pattern recognition molecules mannose-binding lectin (MBL) or ficolin to cell surface carbohydrates such as pneumococcal capsular polysaccharide (Fig. 1). A recent study found that compared to children, older adults are disproportionately affected by pneumococcal serotypes such as 11A and 35B, which interact with L-ficolin (ficolin-2) [10], a component of the lectin pathway (Fig. 1). There have been few dedicated studies of the complement system in older adults, particularly for the lectin pathway, and the consequences of lectin pathway deficiencies are not well understood. There is also a paucity of prior studies of inflammation and the complement system that specifically include older adults who are healthy, without chronic diseases which may influence immune function. Furthermore, measured ficolin levels may be spuriously affected by specimen collection and handling procedures [11] as well as by prolonged storage at − 80 °C [12], which may have influenced the results of previous studies.

## Methods

### Aims and study design

The overall goal of our study was to measure complement pathway components and aging biomarkers among healthy older and younger adults, in order to investigate potential innate immune mechanisms that may underlie aging-related susceptibility to infections.

First, we compared levels of lectin complement pathway components (including L-ficolin) between age groups. Our protocol included meticulous specimen collection and processing techniques to prevent spurious ficolin results. We hypothesized that older adults would have deficiencies in specific lectin pathway components that may account for susceptibility to serotypes of *S. pneumoniae* that bind L-ficolin [10].

We also compared inflammatory markers and classical pathway components (including C1q) levels between younger and older adults, as aging-related biomarkers that could indicate the presence of inflammaging among healthy older adult participants. We hypothesized that older adults would have higher levels of C1q. We also hypothesized that older adults would also have higher levels of inflammatory markers IL-6, TNF-α, and CRP (as previously described in the literature) [4, 6], demonstrating the presence of inflammaging in our healthy older cohort.

### Recruitment of participants

Study-related procedures were approved by the Institutional Review Board of the University of Alabama at Birmingham, and written informed consent was obtained from all participants. Healthy volunteers 19–54 years old, and ≥ 70 years old were included and analyzed in young and older adult groups, respectively. Young participants were recruited from the local community using promotional materials, and older adults were recruited through the geriatrics clinic at the University of Alabama at Birmingham, which posted informational flyers and assisted with identification of potential participants. Potential participants were excluded if they reported any of the following at time of enrollment: diagnosis of chronic obstructive pulmonary disease, active malignancy (other than non-melanoma skin cancer), uncontrolled or severe diabetes mellitus, thyroid or other endocrine disorders, heart/liver/kidney disease, ongoing use of immunosuppressive medications, or smoking history of > 10 pack-years.

### Specimen collection and processing

Blood was collected in glass serum tubes [11] and immediately placed into an insulated transport incubator containing heated wax inserts to maintain temperature of 38 °C as previously described [13]. Following 30 min incubation, samples were immediately transferred to ice. Aliquots of serum were prepared, and kept at − 80 °C prior to analysis. All analysis was completed within 16 months of the date of collection for the first participant.

### Complement and cytokine assays

Assays for complement factors C3 and C4, C-reactive protein (CRP), and classical pathway activity (CH50) were performed at the clinical laboratory at the University of Alabama at Birmingham, using the respective Optilite turbidimetric assay kits (The Binding Site). Assays were performed using the automated Optilite Analyzer according to the manufacturer’s instructions.

Assays for IL-6, tumor necrosis factor-α (TNF-α), and MBL activity were performed in the Nahm Laboratory at the University of Alabama at Birmingham. IL-6 and TNF-α were measured in duplicate using Quantikine High Sensitivity ELISA (R&D Systems) according to the manufacturer’s instructions. Assay ranges were 0.2–10 pg/ml for both IL-6 and TNF-α [14], and these assays demonstrated mean inter-assay coefficient of variation (CV) of 4.6 and 8%, respectively. MBL activity was measured in duplicate using the WIESLAB Complement system MBL pathway enzyme immunoassay kit (Euro Diagnostica) according to the manufacturer’s instructions.

Assays for the concentration of C1q [15], MBL [15, 16], MBL-associated serine proteases 1, 2, and 3 (MASP-1, MASP-2, MASP-3) [15, 17, 18], MBL-associated proteins of 44 and 19 kDa (MAp 44 and MAp 19) [15, 18, 19], M-ficolin (ficolin-1), L-ficolin (ficolin-2), H-ficolin (ficolin-3) [20, 21] and collectin-L1 (CL-L1) [22] were performed as time-resolved immunofluorometric assays at Aarhus University, Denmark as previously described. In brief, samples were incubated in microtiter wells coated with relevant capture antibodies or mannan (for MBL assay) or acetylated bovine serum albumin (for H-ficolin assay). Standards, samples and controls were diluted and added to microtiter wells using a Janus Varispan automated workstation (PerkinElmer). All samples were added in duplicate. In-house biotinylated antibodies, europium-labelled streptavidin (PerkinElmer) and enhancement solution (Ampliqon) were added in successive steps with washing in-between. Bound europium was detected with by time-resolved fluorometry (Victor X5, PerkinElmer). Each microtiter plate contained three quality controls, and the intra- and inter-assay coefficients of variation were below 15% for all assays.

### Statistical analysis

Pearson Chi-square was used to compare race and sex between young and older groups. The primary analysis included comparison of mean levels for each parameter between young and older groups using two sample t-tests. Spearman’s rho was used to evaluate for correlation between inflammatory markers and complement pathway components.

Based on the finding of unequal distribution of race between young and older groups, we subsequently performed a secondary analysis that included adjustment for demographic variables. In the secondary analysis, linear regression models adjusted for sex and race were estimated to determine significant differences in mean levels between age groups. Least squares means of these parameters were estimated and compared between different combinations of demographic factors. P-values of < 0.05 were considered statistically significant for all analyses. Statistical analysis was performed using SPSS version 25 (IBM) and SAS 9.4.

## Results

### Characteristics of study participants

Characteristics of participants are summarized in Table 1. The younger age group consisted of 30 participants with mean age 32.4 ± 10.2 years (range 19–54). The old age group consisted of 27 participants with mean age 76.9 ± 5.3 years (range 70–87). There were 16 females (53.3%) in the young group and 12 (44.4%) in the old group (P = 0.503). Twelve participants (40.0%) in the young group self-reported race as white as compared to 20 participants (74.1%) in the older group (P = 0.010). The remaining participants in each group that self-reported race other than white reported race as either black or Asian. None of the participants reported active smoking at the time of the study, and none had smoking history of > 5 pack-years.

**Table 1:** Characteristics of study participants

	Young (n = 30)	Old (n = 27)	*P*-value
**Mean age, years (SD)**	32.4 (10.2)	76.9 (5.3)	–
**Female, n (%)**	16 (53.3)	12 (44.4)	0.503*
**White, n (%)**	12 (40.0)	20 (74.1)	0.010*

*Pearson Chi-Square

### Measurement of complement pathway components

**Fig. 2:**
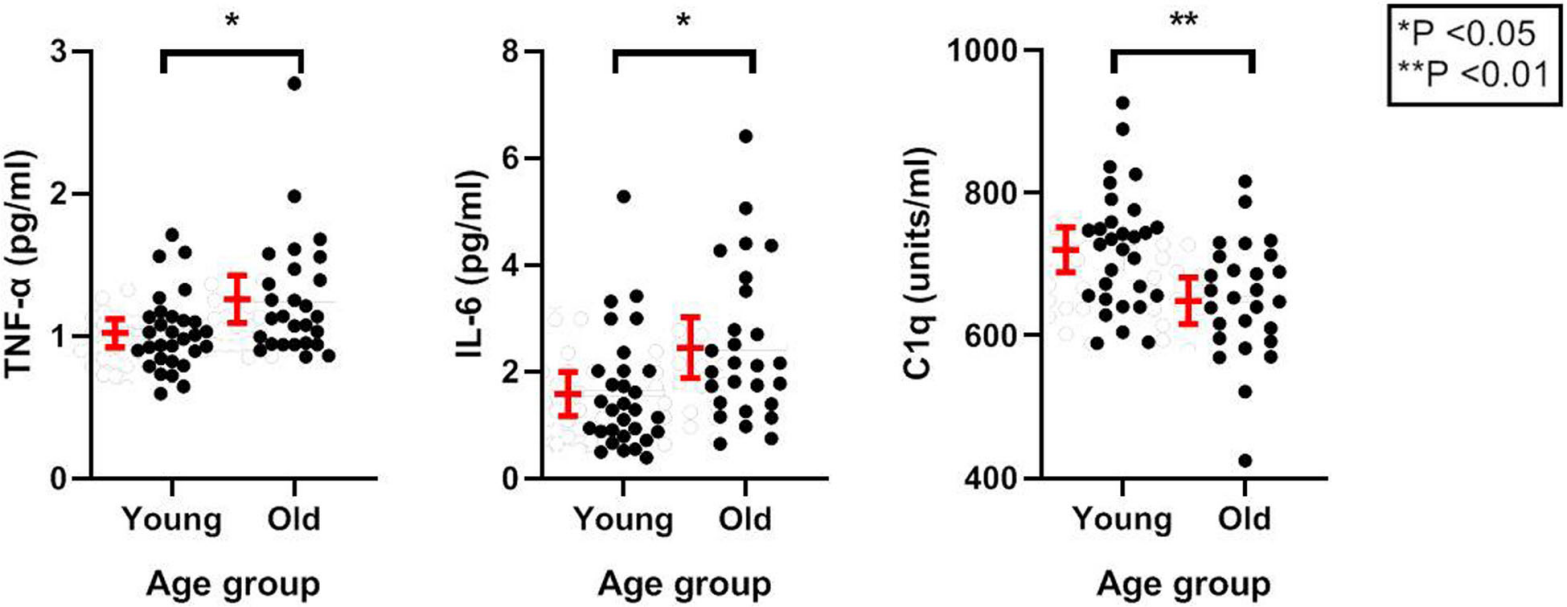
IL-6, TNF-α, and C1q levels in young versus older adults. Comparison IL-6, TNF-α, and C1q levels between younger (ages 18–54) and older (age ≥ 70) adults. Asterisks denote statistically significant differences between groups (independent samples t-test), and red bars indicate mean values with 95% confidence intervals

In unadjusted analysis, mean C1q levels were found to be significantly higher in the younger group (722.7 vs 651.4 units/ml, P = 0.002) (Table 2, Fig. 2). In the linear regression model, there was significant association between race and C1q (P = 0.015), however C1q remained significantly higher in the younger group after adjustment for sex and race (714.8 vs 662.8 units/ml, P = 0.028). Least squares means analysis demonstrated that C1q levels were significantly higher in nonwhite participants compared to white participants in the younger group (750.7 vs 679.0 units/ml, P = 0.022) (Table 3). There were no significant associations between sex and levels of C1q, IL-6, or TNF-α.

**Table 2:** Mean levels of inflammatory markers and complement pathway components in young and old adults

	Young*	Old *	*P*-value
**CRP (mg/L)**	2.37 (2.16)	2.60 (1.91)	0.671
**IL-6 (pg/ml)**	1.59 (1.10)	2.46 (1.44)	**0.013**
**TNF-α (pg/ml)**	1.02 (0.266)	1.26 (0.42)	**0.013**
**CH50 (U/mL)**	69.13 (16.25)	74.10 (15.86)	0.249
**C1q (units/ml)**	722.7 (84.3)	651.4 (82.1)	**0.002**
**C3 (mg/dL)**	131.8 (22.3)	121.1 (26.1)	0.102
**C4 (mg/dL)**	27.40 (8.62)	25.70 (6.92)	0.420
**MBL (ng/ml)**	1650 (1251)	1889 (1541)	0.520
**MBL (% activity)**	45.37 (39.41)	53.78 (39.23)	0.424
**CL-L1 (ng/ml)**	486.2 (71.8)	482.8 (80.6)	0.866
**MASP-1 (ng/ml)**	12,117 (2838)	11,539 (3492)	0.493
**MASP-2 (ng/ml)**	600.1 (366.2)	507.6 (171.8)	0.236
**MASP-3 (ng/ml)**	7184 (1943)	7481 (2097)	0.580
MAp 44 **(ng/ml)**	2127 (519)	2166 (423)	0.758
MAp 19 **(ng/ml)**	372.6 (85.2)	360.1 (96.4)	0.604
**H-ficolin (ng/ml)**	24,091 (9085)	27,370 (5889)	0.116
**M-ficolin (ng/ml)**	3500 (1195)	3813 (1056)	0.304
**L-ficolin (ng/ml)**	3044 (1403)	3314 (995)	0.403

*Mean (standard deviation)

**Table 3:** Least squares means comparisons of IL-6, TNF-α and C1q levels between specific combinations of age and race

Parameter	Groups		*P*-value
**IL-6**	Old	Young	0.006
	Old/nonwhite	Young/white	0.014
	Old/white	Young/white	0.034
**TNF-α**	Old	Young	0.018
	Old/white	Young/white	0.007
**C1q**	Old	Young	0.028
	White	Nonwhite	0.015
	Old/white	Young/nonwhite	< 0.001
	Young/nonwhite	Young/white	0.022

There were no significant differences observed between age groups for other classical or lectin pathway components (Table 2). In the older group, there was a positive correlation between IL-6 and L-ficolin levels (r = 0.49, P = 0.010) and between IL-6 and H-ficolin levels (r = 0.44, P = 0.021). Among older adults, there were significant negative correlations between IL-6 and MBL (r = − 0.396, P = 0.041) and between IL-6 and MBL % activity (r = − 0.441, P = 0.021). In the younger group, there was a positive correlation between IL-6 and M-ficolin levels (r = 0.38, P = 0.04). There were no significant correlations between TNF-α levels and ficolins, or between IL-6 or TNF-α levels and C1q (data not shown).

### Measurement of inflammatory markers

The older age group had significantly higher mean levels of both IL-6 (2.46 vs 1.59 pg/ml, P = 0.013) and TNF-α (1.26 vs 1.02 pg/ml, P = 0.013) (Table 2, Fig. 2). Both IL-6 (P = 0.006) and TNF- α (P = 0.018) remained significantly higher in the older group after adjustment for sex and race. There was no difference in mean CRP levels between groups (P = 0.671).

## Discussion

To our knowledge, our study is the first to perform a between-group comparison of complement components in multiethnic groups of older and younger adults. Our findings contribute to a limited body of existing literature concerning innate immune function in healthy older adults.

We did not observe differences in lectin pathway components to account for susceptibility to ficolin-binding serotypes of *S. pneumoniae* among older adults. We did find significantly higher levels of inflammatory markers among older adults, supporting the presence of inflammaging in this population. However, our finding of higher C1q levels among younger adults was unexpected. Through additional analysis we were able to identify race-related differences in C1q which have not been previously reported. This finding has important implications for further studies of this classical pathway component, which has been proposed as a mediator for normal aging, and identified as a key biomarker for frailty and aging-related disease.

### Lectin pathway

Relatively little is known regarding changes in complement pathways, particularly the lectin pathway, which may be involved with inflammaging and susceptibility to pneumococcal infections. Furthermore, ficolin-2 levels from prior studies may have been falsely low due to the effects of ficolin-2 binding by components of plastic serum tubes [11] or ficolin-2 inhibitors which can arise as a result of prolonged storage [12]. Using meticulous specimen collection and handling methods, our study found no deficiencies in lectin pathway components among older adults to account for increased susceptibility to ficolin-binding serotypes of pneumococcus in this population. Interestingly, we did find significant positive correlations between L and H-ficolin levels and IL-6, and negative correlations between MBL levels/activity and IL-6 among the older adults. This suggests a potential contributory role for ficolin (as opposed to MBL)-mediated lectin pathway activation in inflammaging, and warrants further investigation.

There have been few prior studies of the lectin pathway in aging, and there is very little existing data concerning specific lectin pathway components. Simell et al. previously reported no differences in overall lectin pathway activity between younger and older adults in a Finnish cohort, despite increased classical and alternative pathway activity among similarly aged elderly adults as those in our older age group [23]. Gaya da Costa et al. recently found significantly lower MBL-lectin pathway activity among the oldest 25 participants compared to the youngest 25 participants in a study of 120 healthy Caucasian adults, despite lack of significant association between age and MBL-lectin pathway activity overall [24]. In contrast, our results did not demonstrate significant differences in lectin pathway components between age groups. However, there was a high degree of heterogeneity among individuals in both younger and older adults, and further studies to identify other characteristics associated with these components would be valuable.

The complement system only represents one of the potential processes involved with immune dysregulation in aging. Impaired phagocyte function [23, 25] and/or reduced functional antibody-mediated immune responses [26] among elderly adults could also contribute to increased susceptibility to infections in this population. Further investigation of other pathways involved in inflammation and immune dysfunction with aging are needed in order to better characterize the mechanisms which underlie aging-related disease.

### Inflammatory markers

Inflammatory markers and complement pathway components have been associated with multiple aging-related disease processes, and some have been proposed as biomarkers for frailty and disease. However, the detailed mechanisms which underlie the role of inflammaging in these processes remain incompletely understood. Metabolic dysregulation, innate immune activation, and/or chronic antigen exposure via gut microbiota and cell death have been identified as potential explanations for inflammaging [2], and elevated levels of these markers may reflect low-level immune responses to bacterial pathobionts in the gut or respiratory tract (including pneumococcus).

IL-6 is involved in immune responses to pathogens, and levels have been reported to increase with aging and are often used as a marker of systemic inflammation [4]. It has been shown to predict hospitalization, mortality, and global functional decline in the elderly [6, 27]. Increased IL-6 levels have been associated with disease severity in congestive heart failure [28] and mortality in Parkinson’s disease [29]. As hypothesized, the results of our measurement of inflammatory markers were consistent with prior studies of inflammaging, with higher levels of IL-6 and TNF-α among elderly adults.

### Complement protein C1q

As an initiating factor in the classical pathway, C1q is also involved in immunity and chronic inflammation [30]. C1q has been identified as a potential mediator of aging [31], and elevated C1q has previously been associated with reduced muscle mass [32, 33] as well as neurodegenerative [34] and retinal diseases [35]. Our finding of higher C1q levels among the younger adults was unexpected, since C1q has previously been identified as a potential mediator of normal aging in a mouse model [31], and appears to be involved in neurodegenerative disease among older adults [36, 37]. Prior studies of C1q and aging have yielded variable results. While Watanabe et al. found that C1q was significantly higher among middle-aged/older adults compared to young adults, and that C1q correlated positively with age [33], two other studies found no correlation between C1q levels and age [24, 38].

Our linear regression model identified a significant interaction between race and C1q levels; however, in the adjusted analysis C1q levels remained significantly higher in the younger group. To our knowledge, this association has not been previously reported. Race-related differences in C1q levels must be considered when attempting to compare our results to other studies performed in different populations, and in the design of future studies. The results of our study also have potential implications for the use of C1q as a biomarker. Any attempts to define reference ranges C1q for prognostic or diagnostic use must account for potential race-related differences that may influence classification of results. Applying reference ranges developed in Caucasian study populations could lead to misclassification of C1q levels among nonwhite participants (e.g. false-positive result incorrectly suggesting increased C1q if Caucasian reference ranges were used). Potential genetic basis for variation in C1q has not been previously studied, and warrants further investigation.

It is also possible that factors unaccounted for in our model could also influence C1q levels. Prior studies have reported variation in C1q levels with lifestyle changes such as exercise, body mass index, and time of day [32, 33, 39, 40] as well as systolic blood pressure and high density lipoprotein cholesterol (HDL-C) [38]. We did not collect such data or control for these factors in our study. Cigarette smoke exposure can also result in reduced C1q, which may be involved in the development and progression of emphysema [41]. However, this was unlikely to have affected our results, since individuals with COPD and/or > 10 pack-year smoking history were ineligible for participation, and none of the participants were active smokers at the time of sample collection.

Our enrollment of healthy, relatively fit older adults could potentially account for differences in our findings compared to studies that have primarily studied C1q in the setting of specific degenerative disease processes (particularly in the central nervous system), or may have included elderly participants with chronic diseases, which are very common in this population. Taken within the context of previously-reported disease-specific associations for C1q, our findings emphasize the need to further characterize the role of this important complement component in normal aging as well as in age-related degenerative disease.

### Strengths and limitations

The study of healthy older adults can be logistically challenging, and this group is consequently underrepresented in the literature. We specifically enrolled participants ≥70 years old, and carefully screened potential participants to exclude those with significant chronic diseases that would be likely to influence immune function. While complete medical histories were not available for all older participants, the most common comorbidities were dyslipidemia (7 participants), well-controlled hypertension (6 participants), and well-controlled diabetes mellitus (2 participants). Only one participant was documented to have osteopenia/osteoporosis. BMI among those who had medical records was normal (median = 25; mean = 27). The enrollment procedures sought to recruit a relatively fit group of older adult participants, however standardized measures of fitness or frailty were not available. We also performed meticulous collection, handling, processing, and storage of specimens to prevent spurious ficolin results. As a result of these protocols our sample size was limited, and this should be taken into consideration when attempting to apply these findings to broader populations. However, the novel finding of race-related differences in complement components does provide impetus for additional investigation of inflammaging among different racial/ethnic groups.

## Conclusions

We observed increased levels of inflammatory markers among healthy older adults, consistent with our hypothesis based on the process of inflammaging. While our study of complement pathway components did not indicate differences between age groups that could help explain increased susceptibility to pneumococcal infections among older adults, we observed unexpected and previously unreported race-related differences in complement protein C1q, which has been identified as a potential biomarker for frailty and disease. Our findings have potential implications for future studies, and also emphasize the importance of ongoing investigation of immune dysregulation in aging-related pathology.

## Data Availability

The datasets generated or analyzed during this study are available from the corresponding author upon reasonable request.

## Abbreviations

IL-6: Interleukin-6
TNF-α: Tumor necrosis factor-α
CRP: C-reactive protein
MBL: Mannose-binding lectin
CL-L1: Collectin L1
IgG: Immunoglobulin G
ELISA: Enzyme-linked immunosorbent assay
MASP-1, MASP-2, MASP-3: MBL-associated serine proteases 1, 2, and 3
MAp44: MBL-associated proteins of 44 kDa
MAp19: MBL-associated proteins of 19 kDa
