# Correction to: Classical and lectin complement pathways and markers of inflammation for investigation of susceptibility to infections among healthy older adults

**DOI:** 10.1186/s12979-021-00243-y

**Published:** 2021-08-07

**Authors:** David C. LaFon, Steffen Thiel, Young-il Kim, Mark T. Dransfield, Moon H. Nahm

**Affiliations:** 1 Division of Pulmonary, Allergy, and Critical Care, University of Alabama at Birmingham, Birmingham, AL USA; 2 UAB Lung Health Center, Birmingham, AL USA; 3 Department of Biomedicine, Aarhus University, Aarhus, Denmark; 4 Division of Preventive Medicine, University of Alabama at Birmingham, Birmingham, AL USA; 5 Birmingham VA Medical Center, Birmingham, AL USA; 6 Department of Microbiology, University of Alabama at Birmingham, Bevill Building Room 614 (BBRB 614), 845 19th Street South, Birmingham, AL 35294 USA

**Correction to: Immun Ageing 17, 18 (2020)**

**https://doi.org/10.1186/s12979-020-00189-7**

Following publication of the original article [1], the authors reported an error in the ‘Methods’ section under the subheading entitled ‘Complement and cytokine assays,’ the description of assays should read as follows (change highlighted in bold below):

“Assays for complement factors C3 and C4, C-reactive protein (CRP), and classical pathway activity (CH50) were performed at the clinical laboratory at the University of Alabama at Birmingham, using the respective Optilite **turbidimetric assay** kits (The Binding Site).”

The original article [1] has been updated.
